# Placebo Effect of Caffeine on Substrate Oxidation during Exercise

**DOI:** 10.3390/nu13030782

**Published:** 2021-02-27

**Authors:** Jorge Gutiérrez-Hellín, Carlos Ruiz-Moreno, Millán Aguilar-Navarro, Alejandro Muñoz, David Varillas-Delgado, Francisco J. Amaro-Gahete, Justin D. Roberts, Juan Del Coso

**Affiliations:** 1 Exercise and Sport Science, Faculty of Health Sciences, Universidad Francisco de Vitoria, 28223 Pozuelo, Spain; jorge.gutierrez@ufv.es (J.G.-H.); millan.aguilar@ufv.es (M.A.-N.); alejandro.munoz@ufv.es (A.M.); 2 Exercise Physiology Laboratory, Camilo José Cela University, 28692 Villanueva de la Cañada, Spain; cruizm@ucjc.edu; 3 Faculty of Medicine, Universidad Francisco de Vitoria, 28223 Pozuelo, Spain; david.varillas@ufv.es; 4 Department of Medical Physiology, Faculty of Medicine, University of Granada, 18 Granada, Spain; amarof@ugr.es; 5 Cambridge Centre for Sport and Exercise Sciences, School of Psychology and Sport Science, Anglia Ruskin University, Cambridge CB1 1PT, UK; justin.roberts@aru.ac.uk; 6 Centre for Sport Studies, Rey Juan Carlos University, 28943 Fuenlabrada, Spain

**Keywords:** dietary supplement, ergogenic aid, psychological advantage, carbohydrate

## Abstract

By using deceptive experiments in which participants are informed that they received caffeine when, in fact, they received an inert substance (i.e., placebo), several investigations have demonstrated that exercise performance can be enhanced to a similar degree as a known caffeine dose. This ‘placebo effect’ phenomenon may be part of the mechanisms explaining caffeine’s ergogenicity in exercise. However, there is no study that has established whether the placebo effect of caffeine is also present for other benefits obtained with acute caffeine intake, such as enhanced fat oxidation during exercise. Therefore, the aim of this investigation was to investigate the placebo effect of caffeine on fat oxidation during exercise. Twelve young men participated in a deceptive double-blind cross-over experiment. Each participant completed three identical trials consisting of a step incremental exercise test from 30 to 80% of $\dot{V}$O_2max_. In the two first trials, participants ingested either 3 mg/kg of cellulose (placebo) or 3 mg/kg of caffeine (received caffeine) in a randomized order. In the third trial, participants were informed that they had received 3 mg/kg of caffeine, but a placebo was provided (informed caffeine). Fat oxidation rates were derived from stoichiometric equations. In received caffeine, participants increased their rate of fat oxidation over the values obtained with the placebo at 30%, 40%, 50%, and 60% of $\dot{V}$O_2max_ (all *p* < 0.050). In informed caffeine, participants increased their rate of fat oxidation at 30%, 40%, 50% 60%, and 70% of $\dot{V}$O_2max_ (all *p* < 0.050) over the placebo, while there were no differences between received versus informed caffeine. In comparison to placebo (0.32 ± 0.15 g/min), the rate of maximal fat oxidation was higher in received caffeine (0.44 ± 0.22 g/min, *p* = 0.045) and in informed caffeine (0.41 ± 0.20 g/min, *p* = 0.026) with no differences between received versus informed caffeine. However, the intensity at which maximal fat oxidation rate was obtained (i.e., Fat_max_) was similar in placebo, received caffeine, and informed caffeine trials (42.5 ± 4.5, 44.2 ± 9.0, and 41.7 ± 10.5% of $\dot{V}$O_2max_, respectively, *p* = 0.539). In conclusion, the expectancy of having received caffeine produced similar effects on fat oxidation rate during exercise than actually receiving caffeine. Therefore, the placebo effect of caffeine is also present for the benefits of acute caffeine intake on substrate oxidation during exercise and it may be used to enhance fat oxidation during exercise in participants while reducing any risks to health that this substance may have.

## 1. Introduction

Caffeine is considered as a potent ergogenic aid with the capacity of increasing performance in a wide spectrum of exercise and sports [1,2,3]. For this reason, caffeine is broadly consumed before exercise in doses from 3 to 9 mg per kg of body mass [4] (i.e., mg/kg) in the form of coffee or tea (natural sources) or in capsules/pills, pre-workout supplements, energy drinks, and caffeinated gums (artificial sources) [5]. Although less-well studied, acute caffeine intake can also produce a shifting in substrate oxidation during exercise when ingested in doses similar to the ones used for performance enhancement [6,7]. Briefly, pre-exercise ingestion of caffeine has the capacity to increase fat oxidation rate during incremental [8] and steady-state exercise [9] while it concomitantly reduces carbohydrate utilization. The capacity of caffeine to shift substrate oxidation is habitually found at low-to-moderate exercise intensities, which limits its effectiveness when performing near-to-maximal intensity exercise [6,10]. Although enhanced fat utilization probably adds little to caffeine’s ergogenicity in most sport situations, the utilization of caffeine prior to endurance exercise may benefit those enrolled in exercise programs for body weight loss. Overall, there is ample consensus to consider antagonism on adenosine receptors as the main mechanism behind caffeine’s ergogenicity [11] while the lipolytic action of caffeine may also contribute to its effects on substrate oxidation [12].

Interestingly, several recent investigations have suggested that the belief of having ingested caffeine without actually ingesting the substance, may increase exercise performance [13,14,15,16,17] suggesting that positive expectations of receiving caffeine can add to the pharmacological actions of caffeine [18]. This phenomenon, known as the placebo effect of caffeine, is enabled by the endogenous release of opioids and non-opioids, and it has been found for other active substances [19]. However, two factors are specific for caffeine: first, a high portion of the world population consumes or has consumed caffeine and they have likely experienced the stimulating effects that this substance produces; second the performance-enhancement properties of caffeine are well-known, and individuals expect higher performance when they are told that have ingested caffeine. However, to date, no previous investigation has established whether the placebo effect of caffeine is also present for enhanced fat oxidation during exercise.

The purpose of this experiment was to investigate the placebo effect of caffeine on fat oxidation during exercise. We hypothesized that participants would have increased fat oxidation during exercise when they are told that they had received caffeine. However, we also hypothesized that the placebo effect of caffeine would be lower than the pharmacological effect of acute caffeine ingestion.

## 2. Materials and Methods

### 2.1. Participants

Twelve young male participants volunteered to participate in the study (age = 23 ± 5 years, body mass = 69.0 ± 10.6 kg, height =1.77 ± 0.06 m, body fat = 9.4 ± 3.5%, and maximal oxygen uptake ($\dot{V}$O_2max)_ = 56.2 ± 7.7 mL/kg/min). An a priori sample size calculation indicated that 12 participants were needed to obtain statistically significant differences in maximal fat oxidation rate with three mg of caffeine per kg of body mass respect to the placebo. This a priori sample size was calculated to obtain an effect size of 1.01 in Cohen’s *d* units (statistical power of 80% with type I error set at 5%), based on a previous investigation that obtained this effect when using the same dose of caffeine during an incremental exercise test [8]. The required sample size was determined using G*Power software [20]. The following inclusion criteria were established for potential participants: (a) age between 18 and 40 years; (b) regular exercise training of > 1 h per day, > 4 days per week for the prior six months; and (c) low daily caffeine consumption [21]. The following exclusion criteria were applied to potential participants: (a) lower limb injury within the prior two months; (b) smoking; (c) use of any type of medication or dietary supplement in the previous month; (d) history of cardiopulmonary, metabolic, or musculoskeletal diseases; or (e) allergy to caffeine. Participants were notified in detail of all experimental procedures, potential risks and discomforts associated with the experiment before they signed an informed written consent. The study was approved by the Francisco de Vitoria University Research Ethics Committee (18-2020) and was conducted in accordance with the last version of the Declaration of Helsinki.

### 2.2. Experimental Design

A deceptive, double-blind and cross-over design was used for this investigation. Each participant underwent three trials separated by three-to-five days. The time between trials was set to permit complete physical recovery and substance elimination [22], while reducing the effect of physical training in the experiments (i.e., less than ten days between the first and last trial). In the two first trials, and in a randomized order, participants ingested either 3 mg/kg of body mass of a placebo (cellulose, Guinama, Spain) or 3 mg/kg of body mass of caffeine (Bulk Powders, London, United Kingdom). We selected 3 mg/kg of caffeine because we have recently found that this dose is effective in enhancing fat oxidation during incremental [8] and steady-state exercise [9] in individuals with low habitual caffeine consumption. In the third trial, participants ingested a placebo, but they were informed that they had received 3 mg/kg of caffeine. Initially, participants were informed that they would take part in two experimental trials investigating the effects of caffeine on fat oxidation during exercise. Once the two first two trials were completed, they were told about the need to perform a third trial because the data during the tests with caffeine had not been recorded. Therefore, with this deceptive model, participants had the expectancy of having received caffeine in the third trial. Before the onset of the experiment, participants were informed about the findings of research related to acute effects of caffeine intake on fat oxidation rate through the written informed consent and informal conversations during the recruiting process. This was to create the belief in participants that caffeine has a well-proven capacity to increase fat oxidation during exercise. In all trials, participants ingested an opaque capsule 60 min before the onset of the experimental trials. All tests were performed in a laboratory under controlled ambient conditions (21.7 ± 0.9 °C of dry temperature and 41.4 ± 6.0% of relative humidity).

### 2.3. Pre Experimental Trial

One week before the first trial, participants were weighed to calculate caffeine and placebo dosing and body fat was calculated by bioimpedance (Tanita InnerScan Dual, RD-901BK36, Tokyo, Japan). Then, participants underwent a standardized warm-up that included 10-min at 50 W on a cycle ergometer (Ergoselect 4, Ergoline, Bitz, Germany). Afterwards, participants performed a step incremental exercise test on the cycle ergometer to measure $\dot{V}$O_2max_. The test started with a workload of 75 W and it was set a progressive increase of 25 W each min until volitional fatigue. During the test, participants chose a cadence between 70 and 90 rpm. The test was finished when participants were unable to maintain a cadence above 50 rpm. Oxygen uptake ($\dot{V}$O_2_) and carbon dioxide production (VCO_2_) were measured during the incremental exercise test by using a breath-by-breath gas analyser (Ergostik, Geratherm Respiratory, Bad Kissingen, Germany). The step incremental exercise test was considered maximal when all four end criteria for $\dot{V}$O_2max_ were reached at the end of the tests: (i) $\dot{V}$O_2_ maintenance despite increases in ergometric power, (ii) respiratory exchange ratio above 1.10, (iii) rating of perceived fatigue above 19 points by using the 6–20-point Borg scale [23], and (iv) peak heart rate superior to 90% of age-predicted maximal heart rate (i.e., 220-age). $\dot{V}$O_2max_ was defined as the highest $\dot{V}$O_2_ value obtained during the test, once the data had been averaged by using 15-s intervals. Exercise intensity was normalized in the subsequent experimental trials by using 10% increases of $\dot{V}$O_2max_. For this normalization, a regression analysis was performed for each participant for the relationship between wattage and $\dot{V}$O_2_ obtained in this pre-experimental test.

### 2.4. Experimental Protocol

Twenty-four hours before each experimental trial, participants refrained from exhausting exercise and adopted a food intake and fluid intake regimen as they are would do before a sport competition. Fluid and diet guidelines were provided to increase the likelihood of carbohydrate availability [24] and euhydration [25] before all experimental trials. Participants were also required to avoid alcohol, caffeine, and other stimulants for 24 h before each trial. To produce a within-subject standardization of diet, participants completed a 24-h dietary record before the first trial, and they replicated the same dietary pattern before the second and third trials. In each trial, participants were encouraged to abstain from any food for >8 h before the trial (last meal was dinner at 21.00 the night before) and they arrived at the laboratory in a fasted state. Participants were told of the need for drinking ~7 mL/kg of water in the two-hour period before arriving to the laboratory (08.00 am) to assure euhydration. Upon arrival, a urine sample was obtained to assess euhydration, which was confirmed by a urine specific gravity (MASTER-S28M, Atago Company, Tokyo, Japan) < 1.020 [26]. Then, the capsule with the assigned experimental treatment was consumed with 150 mL of tap water while an experimenter certified this process. After that, participants rested for 1 h to permit substance absorption. In the last five minutes of the resting period, heart rate (Wearlink, Polar, Kempele, Finland) and systolic and diastolic blood pressure (M6 Comfort, Omron, Kyoto, Japan) were measured while the participants lay on a stretcher. Then, participants completed a 10-min standardized warm-up at a workload equivalent to 30% of $\dot{V}$O_2max_, as measured in the pre experimental session (Ergoselect 4, Ergoline, Bitz, Germany). Once the warm-up was completed, participants were told that the exercise test had started, and they were not allowed to speak during the whole trial (unless an emergency occurred). The experimental test consisted of a step incremental exercise with 10% $\dot{V}$O_2max_ increases every 3-min. The test finalized once participants had completed the workload equivalent to 80% of $\dot{V}$O_2max_ because the respiratory exchange ratio was above 1.00 in all participants. At the end of each stage, participants were asked about their perceived fatigue by using the 6–20-point Borg scale. Breath-by-breath gas exchange data were collected continuously during the test to measure $\dot{V}$O_2_ and $\dot{V}$CO_2_ (Ergostik, Geratherm Respiratory, Bad Kissingen, Germany). During the last minute of each stage, participants were instructed to maintain a stable position and cadence and gas exchange data and heart rate were averaged to achieve a representative value for each exercise intensity level. At each stage, energy expenditure and fat and carbohydrate oxidation rates were calculated with stoichiometric calculations [27,28] by using $\dot{V}$O_2_ and $\dot{V}$CO_2_. Energy expenditure (in kcal/min) was calculated as (3.869 × $\dot{V}$O_2_) + (1.195 × $\dot{V}$CO_2_), where $\dot{V}$O_2_ and $\dot{V}$CO_2_ are in L/min. Fat oxidation rate (in g/min) was calculated as (1.67 × $\dot{V}$O_2_) − (1.67 × $\dot{V}$CO_2_) and carbohydrate oxidation rate (in g/min) was calculated as (4.55 × $\dot{V}$CO_2_) − (3.21 × $\dot{V}$O_2_). Maximal fat oxidation rate was established for each participant as the maximum value of fat oxidation rate obtained at any stage during the incremental exercise test [29]. The exercise intensity that produced maximal fat oxidation rate was established as Fat_max_. Both maximal fat oxidation rate and Fat_max_ were individually calculated for each participant in each trial by two investigators blinded to the treatments. The position of the saddle and handlebar in the cycle ergometer, cadence in all stages, and the clothing used was replicated in all trials. At the end of the first two experimental trials, participants were asked to guess in which trial they had received caffeine.

### 2.5. Statistical Analysis

The results of each test were introduced into the statistical package SPSS v 22.0 (IBM Corp., Armonk, NY, USA) and analyzed by a researcher blinded to the treatments. The normality of each variable was initially tested with the Shapiro–Wilk test, and, consequently, parametric statistics tests were used to determine differences among trials. The data are presented as mean ± standard deviation. A one-way analysis of variance (ANOVA) was used to detect differences in maximal fat oxidation rate, Fat_max_, and the values obtained at test (i.e., heart rate, blood pressure, and urine specific gravity). A two-way analysis of variance (substance × exercise intensity) was used to determine difference in the variables obtained during the step incremental exercise test. After a significant F test was obtained, differences were identified by Bonferroni post-hoc tests. In all statistical tests, a level of *p* < 0.050 was set to establish statistically significant differences.

## 3. Results

### 3.1. Pre-Exercise Heart Rate, Blood Pressure and Urine Specific Gravity

There was no main effect of substance on resting heart rate, blood pressure variables, or urine specific gravity (Table 1).

### 3.2. Substrate Oxidation, Energy Expenditure, Heart Rate, and Exertion during the Step Incremental Test

**Figure 1:**
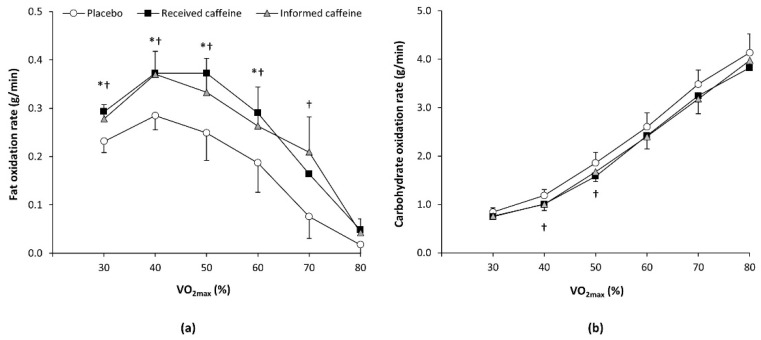
(**a**) Fat oxidation rate and (**b**) carbohydrate oxidation rate during an incremental step exercise test on a cycle ergometer in active individuals who ingested (1) a placebo; (2) three mg of caffeine per kg of body mass (received caffeine); and (3) a placebo, but they were told they were given three mg of caffeine per kg mass (informed caffeine). (*) Received caffeine different from placebo for the same workload at *p* < 0.050. (†) Informed caffeine different from placebo for the same workload at *p* < 0.050.

For the two first trials, only five out of 12 participants (41.7%) correctly guessed when they had received caffeine. There were main effects of substance (F = 4.43, *p* = 0.024) and exercise intensity (F = 17.06, *p* < 0.001) on the rate of fat oxidation during exercise, with a statistically significant interaction between these two factors (F = 2.34, *p* = 0.015). The post-hoc analysis revealed that “received caffeine” trial increased the rate of fat oxidation over the values obtained with the placebo at 30%, 40%, 50%, and 60% of $\dot{V}$O_2max_ (all *p* < 0.050; Figure 1). The “informed caffeine” trial increased the rate of fat oxidation by 30%, 40%, 50% 60%, and 70% of $\dot{V}$O_2max_ (all *p* < 0.050), while there were no differences between received caffeine and informed caffeine. For carbohydrate oxidation rate during exercise, there was a main effect of exercise intensity (F = 127.99, *p* < 0.001) and a substance × intensity interaction (F = 2.29, *p* = 0.048). The post-hoc analysis revealed that only informed caffeine reduced carbohydrate oxidation rates at 40% and 50% of $\dot{V}$O_2max_ (all *p* < 0.050).

**Figure 2:**
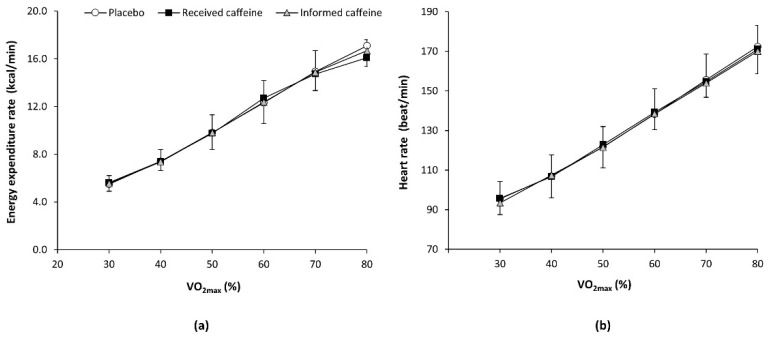
(**a**) Energy expenditure rate and (**b**) heart rate during an incremental step exercise test on a cycle ergometer in active individuals who ingested (1) a placebo; (2) three mg of caffeine per kg of body mass (received caffeine); and (3) a placebo, but they were told they were given three mg of caffeine per kg mass (informed caffeine).

There was a main effect of exercise intensity (F = 480.58, *p* < 0.001) and a substance × intensity interaction (F = 2.49, *p* = 0.039) in the rate of energy expenditure. However, the post-hoc analysis did not reveal any statistically significant difference in the pairwise comparison between experimental trials at any workload. For heart rate, there was only a main effect of exercise intensity (F = 95.85, *p* < 0.001) with no post-hoc differences (Figure 2).

**Figure 3:**
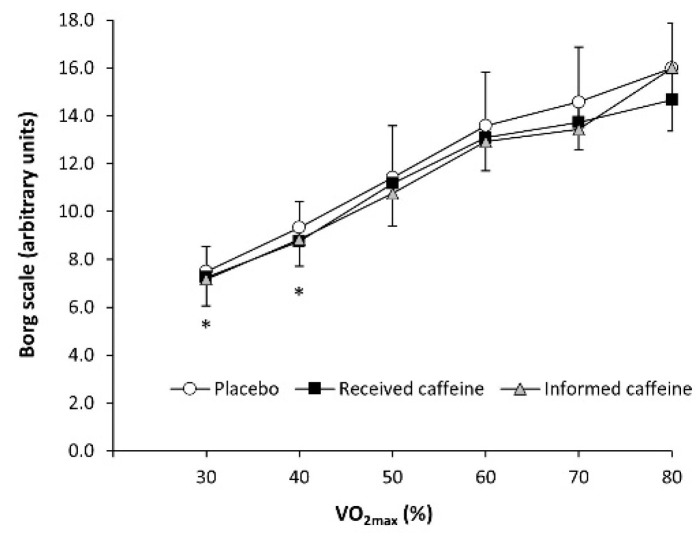
Rating of perceived exertion, measured with the Borg scale during an incremental step exercise test on a cycle ergometer in active individuals who ingested (1) a placebo; (2) three mg of caffeine per kg of body mass (received caffeine); and (3) a placebo, but they were told they were given three mg of caffeine per kg mass (informed caffeine). (*) Received caffeine different from placebo for the same workload at *p* < 0.050.

There were main effects of substance (F = 5.72, *p* = 0.008) and exercise intensity (F = 35.95, *p* < 0.001) in the rating of perceived exertion during exercise, although the interaction between these two factors did not reach statistical significance (F = 1.56, *p* = 0.189). The post-hoc analysis revealed that “received caffeine” decreased the rating of perceived exertion at 30% and 40% of $\dot{V}$O_2max_ (all *p* < 0.050; Figure 3) with no other differences in the remaining workloads between experimental trials.

### 3.3. Maximal Fat Oxidation Rate and Fat_max_

**Figure 4:**
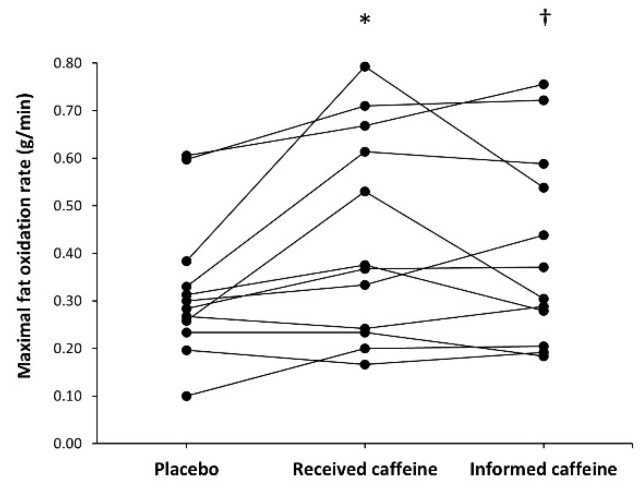
Individual responses for maximal fat oxidation rate during an incremental step exercise test on a cycle ergometer in active individuals who ingested (1) a placebo; (2) three mg of caffeine per kg of body mass (received caffeine); and (3) a placebo, but they were told they were given three mg of caffeine per kg mass (informed caffeine). Each dot represents one participant. (*) Received caffeine different from placebo at *p* < 0.050. (†) Informed caffeine different from placebo at *p* < 0.050.

There was a main effect of substance on maximal fat oxidation rate during exercise (F = 6.31, *p* = 0.007). In comparison to placebo (0.32 ± 0.15 g/min), the rate of maximal fat oxidation was higher in received caffeine (0.44 ± 0.22 g/min, *p* = 0.045) and in informed caffeine (0.41 ± 0.20 g/min, *p* = 0.026). From the total, six participants obtained the highest value of maximal fat oxidation in the trial with received caffeine, five participants in the informed caffeine trial and one participant in the placebo trial (Figure 4). However, there was no main effect of substance on Fat_max_ (F = 0.66, *p* = 0.539) and Fat_max_ value was similar in the placebo, received caffeine, and informed caffeine trials (42.5 ± 4.5, 44.2 ± 9.0, and 41.7 ± 10.5% of $\dot{V}$O_2max_, respectively).

## 4. Discussion

The purpose of the current study was to determine the existence of the placebo effect of caffeine on fat oxidation during exercise. This was based on previous investigations that have found a placebo effect of caffeine on exercise performance [13,14,15,16,17] and have suggested that the expectancy of having received caffeine may induce a placebo-induced performance-enhancement. The main outcomes of this investigation confirm the existence of the placebo effect of caffeine on fat oxidation during exercise as participants increased fat oxidation to a similar extent when they received three mg/kg of caffeine and when they were informed that they had received this dose of caffeine, although a placebo was administered (Figure 1). Specifically, both trials, received caffeine and informed caffeine, increased the rate of fat oxidation over the placebo when exercising at 30%, 40%, 50%, and 60% and the magnitude of the change in maximal fat oxidation was comparable. Additionally, Figure 4 reveals that the pharmacological and placebo effect of caffeine on maximal fat oxidation rate was present in most of the individuals of this sample (11 out of 12), suggesting that these effects can be widely found in recreational male individuals. Collectively, this investigation indicates that the expectancy of having received caffeine was equally effective to the real administration of caffeine on fat oxidation during exercise and suggests that the placebo effect of caffeine may be used to produce a higher utilization of fat during exercise under certain circumstances.

The placebo effect is a phenomenon that occurs when an inert substance is given instead an active medication, but individuals are convinced that they had received the active substance. This belief habitually induces positive outcomes that are attributable to the psychosocial context and individual treatment expectations [19] which creates drug-like effects induced by the release of opioids and nonopioids. The placebo effect of caffeine has been reported in the literature as a performance-enhancing strategy for several forms of exercise such as endurance performance [15,16], muscle performance [13], anaerobic performance [14], and skill-specific sport situations [30], although the placebo effect of caffeine has not been found in all investigations on this topic [17,31]. In most of these investigations, researchers have used deceptive experiments where participants are informed of that they had received an ergogenic dose of caffeine to induce expectancy when in fact, they were administered a placebo. The performance obtained in this trial (i.e., informed caffeine) is habitually compared to a real placebo/control situation and to a trial where participants actually received caffeine (i.e., received caffeine) [32]. However, recent investigations [15,30] have introduced more complex experiments where expectancy and the physiological effect of caffeine are dissociated by using four trials combining informed caffeine/received caffeine, informed caffeine/received placebo, informed placebo/received caffeine, and informed placebo/received placebo. In these latter investigations, it was not clearly observed that combining expectancy with caffeine intake produced a synergistic action on exercise performance.

The current experiment followed a two-phase protocol to induce expectancy as the effect of caffeine to enhance fat oxidation is less well popular than the effect of caffeine on exercise performance. We used such an approach as the knowledge of the potential effect of the substance is essential to induce an effective placebo effect. Initially, we set a double-blinded, randomized, and placebo-controlled experiment with two trials, in which the efficacy of acute caffeine intake (received caffeine) to enhance fat oxidation during an incremental step exercise was compared to a placebo trial, following a previous investigation [8]. We used this initial phase to confirm the physiological effect of caffeine on fat oxidation in our participants, as in these two trials participants did not receive any information about the order of the substances ingested. In addition, the physiological effect of caffeine was effectively isolated from the placebo effect as only 41.7% of the participants correctly guessed when they had received caffeine. The second phase of the experiment included a third trial where participants were told that they were given caffeine, but a placebo was administered (informed caffeine). This phase was conducted after the first phase to assure that participants have experienced the use of caffeine during low-to-moderate exercise intensity, with the ultimate purpose of inducing a real expectancy about caffeine-effect on fat oxidation. In addition, with this two-phase approach, participants were unaware of the objective of the investigation and therefore, they were not conditioned to guess what substance they had ingested in the third trial. In this experiment, we found that the physiological effect of caffeine to increase fat oxidation rate during exercise was similar to the placebo effect of caffeine on this variable, induced by the expectancy of having received caffeine. These data confirm the existence of a psychological-induced effect on fat utilization during exercise when participants believe that they had ingested caffeine.

The mechanism(s) explaining higher fat oxidation in the informed caffeine trial vs. placebo trial is/are not evident from our investigation. However, previous investigations have suggested various mechanisms that may explain the findings of this study. First, it has been speculated that the placebo effect on exercise performance may be the result of participants adopting a more stable pacing strategy when they believed that had received an active treatment [32]. Nevertheless, this mechanism seems not applicable to the current experiment as the exercise workload and cadence were replicated in all trials. The most probable explanation for the increased fat oxidation in the informed caffeine trial may be related to neurobiological mechanisms associated with the placebo effect and expectancy. The placebo effect has been associated with opioid, endocannabinoid, serotonin, and dopamine systems while the most common view is that various neurotransmitter systems are in fact involved at the same time in the placebo effect [33]. This especially applies to the placebo effect of caffeine, as the main effect of this substance on the brain is to block adenosine receptors, resulting in higher concentrations of serotonin, dopamine, and norepinephrine [34]. Additionally, it has been suggested that the placebo effect often activates the same pathways as the drug that it purports to be [35], suggesting then the placebo effect of caffeine may increase the concentration of the same neurotransmitters as the actual ingestion of caffeine. In this regard, the endogenous opioids and endocannabinoid system are implicated in pain mechanisms and analgesia, the serotonin system is associated with anxiety while dopamine has been investigated in the context of motivation [32]. Therefore, it is possible that the expectancy of having received caffeine produces lower pain and anxiety and enhances motivation during exercise. As the brain produces an effective control mechanism to regulate exercise intensity, fatigue, and even metabolism [36], the change in the level neurotransmitters induced by the placebo effect of caffeine may entail neurobiological mechanisms that facilitates changes in substrate oxidation. At this stage, this is a speculation that needs confirmation in further experiments.

The placebo effect of caffeine on fat oxidation during exercise was accompanied by other interesting placebo-induced changes. There was a substance × intensity interaction for carbohydrate oxidation rate (Figure 1). However, the only statistically significant differences in the pairwise comparison to the placebo trial were obtained in the informed caffeine trial at 40 and 50% of $\dot{V}$O_2max_. Additionally, the three trials produced similar values for energy expenditure and heart rate across the exercise intensity range used in this investigation, although perceived exertion at low exercise intensities was only reduced with the actual ingestion of caffeine (Figure 3). This means that participants did not feel reduced fatigue in informed caffeine, despite this being a habitual physiological effect of the placebo effect of caffeine [37]. All this information suggests that the placebo effect may induce additional changes to enhanced fat oxidation during exercise of low-to-moderate intensity such as reduced carbohydrate utilization, while the shifting in substrate oxidation is evident without measurable effect on energy expenditure, heart rate, or perceived fatigue.

The deceptive experimental design used for this investigation has several limitations. The first two experimental trials were performed in a double-blinded and randomized fashion and the percentage of participants who correctly guessed when they had received caffeine suggests that the blinding procedure was successful. However, the informed caffeine trial was always performed after these two initial trials. This was selected to generate a protocol that undoubtedly produced expectancy of receiving caffeine and the potential belief in caffeine-induced effects on substrate oxidation, but this may have generated an order effect in the outcomes of this investigation. Second, the current investigation was performed during an incremental step test in recreationally active male participants. Therefore, the outcomes of this investigation can only be translated to male individuals during exercise of increasing intensity. The confirmation of the placebo effect of caffeine in enhancing fat oxidation during exercise has to be tested in women, during steady state exercise, and in athletes with a higher training background. Additionally, participants in this study were individuals with a low level of daily caffeine consumption. It is probable that the placebo effect of caffeine on fat oxidation during exercise is lower or even inexistent in habitual caffeine consumers, as has been recently found for muscle performance [17]. Third, a recent meta-analysis indicated that the ability of caffeine to increase fat oxidation during exercise is higher in doses ≥ 6 mg/kg when compared to ~3 mg/kg [6]. Future investigations should determine if the expectancy of having received a dose of 6 mg/kg of caffeine also produces a higher effect on fat oxidation during exercise than the expectancy of receiving 3 mg/kg. Fourth, the trials received caffeine and informed caffeine established, independently, the physiological effect of caffeine and expectancy of receiving caffeine, respectively. However, it is still possible that both effects can have a synergistic action when participants are given caffeine and they are told that they receive caffeine (i.e., received caffeine + informed caffeine). Further investigations with this experimental approach are needed to confirm this hypothesis, although it seems that combining the physiological effect of caffeine and expectancy does not produce a synergistic ergogenicity [15], or the effect is slightly higher than the isolated effects of these two strategies [38]. Last, no biological markers relating to mechanistic actions associated with the placebo effect of caffeine were measured. Recently, it has been proposed that that mechanisms associated with the placebo effect of caffeine may mimic real mechanisms associated to acute caffeine intake [32]. Therefore, the measurement of plasma glycerol and free fatty acid concentration [39], and muscle oxygen saturation [40] in future investigations may help to determine the mechanism associated with the placebo effect of caffeine on fat oxidation during exercise. Finally, the current investigation demonstrates the existence of the placebo effect of caffeine on fat oxidation during exercise when the individuals believed that they had ingested a dose of 3 mg/kg of caffeine.

## 5. Conclusions

The findings of the current investigation suggest that expectancy of having received caffeine produces similar effects on fat oxidation rate during exercise to receiving caffeine. Therefore, the placebo effect of caffeine is also present for the benefits of acute caffeine intake on substrate oxidation during exercise. From a practical perspective, the use of the placebo effect of caffeine may enhance fat oxidation during exercise in participants who are aware of the benefits of caffeine but reduce any risks to health that this substance may have [41]. This may be particularly useful in reducing typical side effects associated with caffeine in an exercise setting such as insomnia and nervousness [42]. However, future investigations are needed to determine if the placebo effect of caffeine on fat oxidation can be obtained chronically, as it is probable that expectancy of receiving caffeine can be reduced when an inert substance is administered for several days.

## Figures and Tables

**Table 1:** Variables obtained at test in individuals who ingested (1) a placebo; (2) three mg of caffeine per kg of body mass (received caffeine); and (3) a placebo, but they were told they were given three mg of caffeine per kg mass (informed caffeine).

Variable (Units)	Placebo	Received Caffeine	Informed Caffeine	*p*
Resting heart rate (beat/min)	53 ± 8	52 ± 8	53 ± 9	0.509
Systolic blood pressure (mmHg)	116 ± 12	119 ± 9	117 ± 9	0.639
Diastolic blood pressure (mmHg)	66 ± 10	68 ± 7	66 ± 6	0.191
Urine specific gravity	1.013 ± 0.006	1.014 ± 0.005	1.017 ± 0.003	0.271

## Data Availability

The data presented in this study are available on request from the corresponding author. The data are not publicly available due to legal restrictions.
